# DOSE-L1000: unveiling the intricate landscape of compound-induced transcriptional changes

**DOI:** 10.1093/bioinformatics/btad683

**Published:** 2023-11-11

**Authors:** Junmin Wang, Steven Novick

**Affiliations:** Data Sciences and Quantitative Biology, Discovery Sciences, Biopharmaceuticals R&D, AstraZeneca, Gaithersburg, MD 20878, United States; Global Statistical Sciences, Eli Lilly, Indianapolis, IN 46285, United States

## Abstract

**Motivation:**

The LINCS L1000 project has collected gene expression profiles for thousands of compounds across a wide array of concentrations, cell lines, and time points. However, conventional analysis methods often fall short in capturing the rich information encapsulated within the L1000 transcriptional dose–response data.

**Results:**

We present DOSE-L1000, a database that unravels the potency and efficacy of compound-gene pairs and the intricate landscape of compound-induced transcriptional changes. Our study uses the fitting of over 140 million generalized additive models and robust linear models, spanning the complete spectrum of compounds and landmark genes within the LINCS L1000 database. This systematic approach provides quantitative insights into differential gene expression and the potency and efficacy of compound-gene pairs across diverse cellular contexts. Through examples, we showcase the application of DOSE-L1000 in tasks such as cell line and compound comparisons, along with clustering analyses and predictions of drug–target interactions. DOSE-L1000 fosters applications in drug discovery, accelerating the transition to omics-driven drug development.

**Availability and implementation:**

DOSE-L1000 is publicly available at https://doi.org/10.5281/zenodo.8286375.

## 1 Introduction

Gene expression signatures play a pivotal role in illuminating the intricate landscape of cellular states, providing invaluable insights into biological processes and disease mechanisms. However, the acquisition of comprehensive gene expression profiles has traditionally been hindered by the limitations of cost and scalability inherent in conventional profiling methods. The Library of Integrated Network-based Cellular Signatures (LINCS) project heralded a new era in this field, offering a groundbreaking approach to elucidate gene expression patterns across a vast array of biological perturbations (Lamb *et al.* 2006, Subramanian *et al.* 2017).

Central to the LINCS project is the utilization of the L1000 technology, a cost-effective platform that enables the measurement of gene expression across an expansive repertoire of cell lines and experimental conditions (Peck *et al.* 2006, Subramanian *et al.* 2017). Notably, the L1000 technology assesses the expression levels of 978 genes in each experimental context, presenting an unparalleled opportunity to interrogate the multidimensional landscape of transcriptional changes.

LINCS L1000 gene expression data hold immerse potential in guiding drug development and repurposing efforts (Iskar *et al.* 2010, Wang *et al.* 2013). Traditionally, evaluating compound-induced transcriptional changes from the L1000 data has relied upon *z*-scores, moderated *z*-scores, the characteristic direction method, and LIMMA (Lamb *et al.* 2006, Clark *et al.* 2014, Ritchie *et al.* 2015, Duan *et al.* 2016, Subramanian *et al.* 2017). *Z*-scores standardize gene expression values to measure their deviation from the mean, and moderated *z*-scores weight replicates based on the correlation between replicate signatures to improve the reliability of the *z*-score calculation (Subramanian *et al.* 2017). The characteristic direction method correlates gene expression changes to predefined direction vectors, and LIMMA borrows strength across genes to enhance the standard deviation estimate and boost testing degrees of freedom, helping to overcome the sample size limitation of many studies (Clark *et al.* 2014, Ritchie *et al.* 2015). While these methods offer valuable means of comparing gene expression changes between conditions, they typically treat each concentration as a separate entity. This approach can potentially overlook the holistic trends and nuances that emerge across multiple doses (Ji *et al.* 2009, House *et al.* 2017).

In addition, the expression profiles of both direct and secondary targets, as well as off-targets can serve as valuable readouts/endpoints for therapeutic interventions. For example, the expression of p21 has proven to be an effective indicator for the efficacy of chemotherapy (Nakamura *et al.* 2004). Characterizing the activity of compound-gene pairs can inform compound selection through the assessment of the relative potency of compounds acting on the same gene. Moreover, it provides a means to inform therapeutic windows by discerning the dose-dependent responses of different on-targets and off-targets to the same compound. However, to our knowledge, no prior research has comprehensively characterized the efficacy and potency of compound-gene pairs in the L1000 database. These oversights underscore the need for a more robust quantitative evaluation of gene expression data in a dose-centric manner.

In this study, we introduce a novel approach that leverages generalized additive models (GAMs) and robust linear models (RLMs) to interrogate compound-induced transcriptional changes (Venables *et al.* 2002, Wood 2017). The focus of our work is to understand how a compound’s exposure affects the level of expression of the target gene. While sigmoidal curves are commonly used and find strong support within the realm of biochemical kinetics (Wang *et al.* 2019, Kiwimagi *et al.* 2021), they may not capture all nonlinear relationships inherent in transcriptional dose–response data, such as Hook effects. In contrast, GAMs not only offer a more versatile and adaptable framework to facilitate streamlined model fitting but also maintain robustness through effective regularization techniques (Hastie and Tibshirani 1999, Benedetti and Abrahamowicz 2004, Wood 2017). In scenarios with limited numbers of doses (≤4), our approach uses RLMs in place of GAMs to ensure robust model fitting by effectively downweighting outliers (Huber 1981, Venables *et al.* 2002). This dual strategy enhances the comprehensiveness of our analysis. Our approach not only robustly reveals the patterns of differential gene expression but also elucidates the potency and efficacy of compound-gene pairs across a wide array of cellular contexts.

Building upon this foundation, we introduce DOSE-L1000, a pioneering database that serves as a repository for the multifaceted results derived from our modeling framework. The novel aspects of this database include (i) a thorough examination of efficacy and potency of compound-gene pairs, and (ii) a comprehensive evaluation of compound-induced gene expression changes using robust statistical methods based on gene expression data within the L1000 database. Our database empowers researchers to explore and decipher the molecular underpinnings of compound-induced effects across a spectrum of concentrations, cell lines, and time points. In presenting our approach and database, we aim to provide a transformative framework for extracting nuanced insights from the wealth of data offered by the LINCS L1000 project, thereby advancing our comprehension of cellular responses and therapeutic interventions.

## 2 Materials and methods

LINCS L1000 quantile-normalized log_2_-transformed expression data, also known as Level 3 data, were acquired from the Gene Expression Omnibus (GEO) data repository and imported into the R programming environment through the cmapR R package (see Supplementary Table S1 for the list of files downloaded) (Edgar *et al.* 2002, Natoli 2023). We used a robust model-based approach to discern the differential expression patterns, as well as the potency and efficacy of compound-gene pairs across diverse cellular contexts. This framework leverages GAMs and RLMs, strategically chosen based on the characteristics of the data under consideration.

### 2.1 Generalized additive models

**Figure 1.**
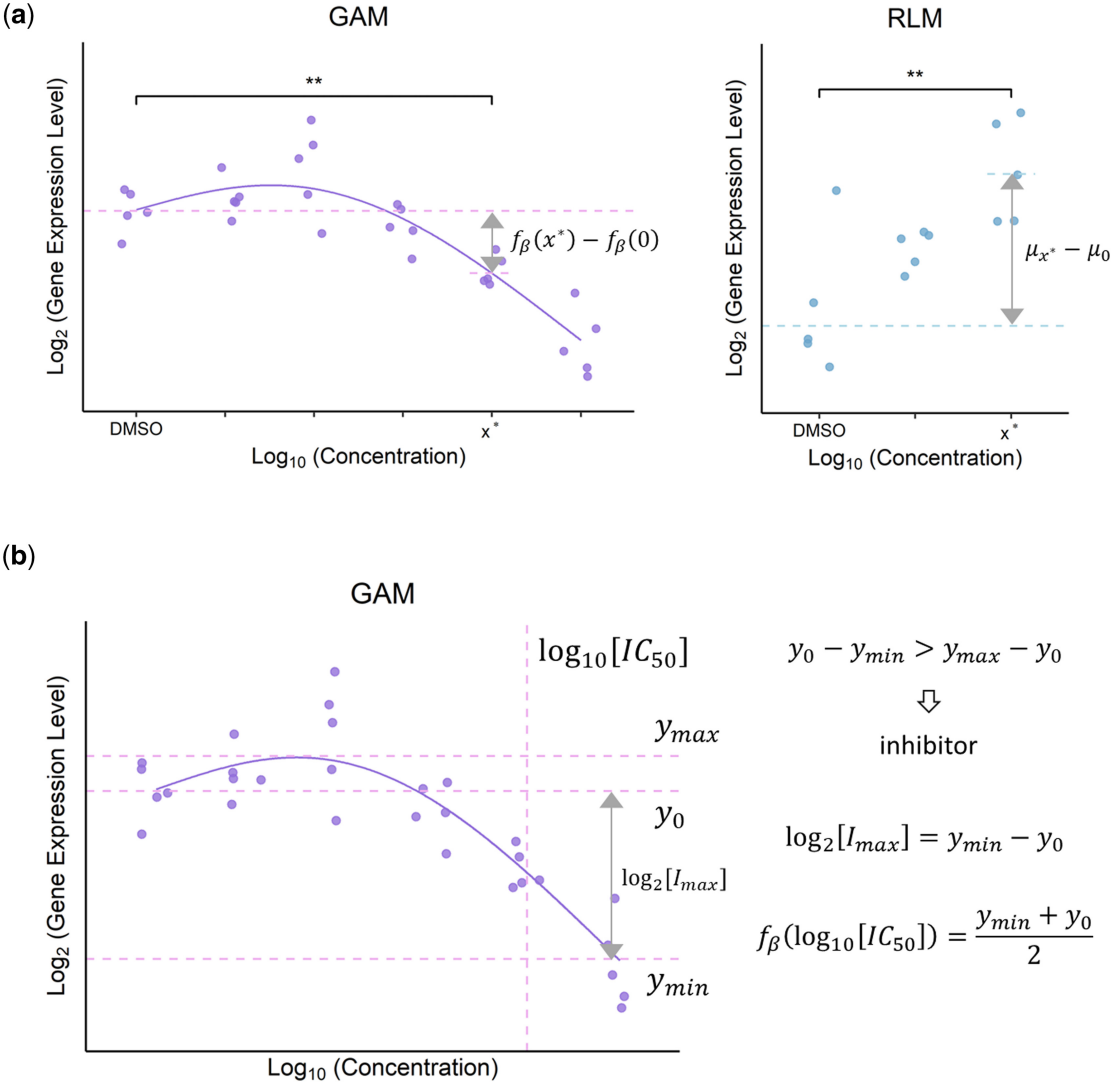
Illustration of a model-based approach for unveiling gene expression signatures, efficacy, and potency. (a) Schematic overview of model fitting and differential expression analysis. Depending on the number of concentrations tested, either GAM or RLM was fitted to the data. Colored dots represent observations, the solid curve depicts the best-fit GAM, and dashed lines represent fitted log_2_ expression levels of the target gene. *T*-tests as described in Section 2.3 are subsequently carried out to identify differentially expressed genes for each nonzero concentration relative to the vehicle plate control. Genes with adjusted *P*-values <0.05 are highlighted by double asterisks. (b) Definitions of $\log_{2}\left[I_{\max}\right]$ and $\log_{10}\left[{IC}_{50}\right]$ for an inhibitor. $\log_{2}\left[I_{\max}\right]$ is defined as the maximum log_2_ fold decrease across tested concentrations ($y_{\min}-y_{0}$), while $\log_{10}\left[{IC}_{50}\right]$ is defined as the minimal log_10_-transformed concentration at which the log_2_ expression level of the target gene reaches the midpoint between that of the vehicle plate control ($y_{0}$) and the minimum log_2_ expression level ($y_{\min}$). Similar definitions apply to $\log_{2}\left[E_{\max}\right]$ and $\log_{10}\left[{EC}_{50}\right]\;$for an activator.

When dealing with a substantial number of concentrations (C>4) and one or more time points (K≥1), we harnessed the flexibility of GAMs to capture the nonlinear nature of transcriptional dose–response relationships (Fig. 1a) (Wood 2017). For cases where K equals 1, the model takes the form:

#(1) yi=fβxi+ϵi,

where $y_{i}$ represents the log_2_-transformed gene expression level in the *i*th sample, $x_{i}$ is the log_10_-transformed compound concentration, and $\epsilon_{i}$ is the normally distributed error term. The smoothing spline function $f_{\beta }$ is expressed as:

#(2) fβxi=bxiβ.

Here, $b\left(x_{i}\right)$ comprises a collection of basis functions that construct the spline, i.e. $b\left(x_{i}\right)=\left[\begin{array}{cccc} b_{1}\left(x_{i}\right) & b_{2}\left(x_{i}\right) & \cdots & b_{J}\left(x_{i}\right) \end{array}\right]$, and β represents the coefficient vector to be estimated, i.e. $\beta ={\left[\begin{array}{cccc} \beta_{1} & \beta_{2} & \cdots & \beta_{J} \end{array}\right]}^{T}$, where J denotes the dimension of the basis.

When K is >1, the model incorporates a dummy variable $t_{ik}$ to differentiate functions across different time levels:

#(3) yi=∑k=1Kfβkxitik+ϵi,

where $t_{ik}$ represents the *k*th time level, and $f_{\beta k}\left(x_{i}\right)$ encompasses the collection of smoothing splines that share a common basis:

#(4) fβkxi=bxiβk.

Here, $\beta_{k}$ represents the coefficient vector corresponding to the kth time level.

To fit GAMs to the data, we used the gam() function within the mgcv R package (Wood 2017). The estimation of f and $f_{k}$ utilized thin plate splines with a 4D basis (Wahba 1990). The choice of basis, along with the decision to treat time as a categorical factor within the model, was driven by the number of concentrations and time points available in the dataset (Supplementary Fig. S1). In addition, we added a pseudo-concentration to the baseline to address the challenge of log-transforming zero values (see Supplementary Table S2 for details).

### 2.2 Robust linear models

We adopted a different approach in instances where the number of concentrations is limited (C≤4). Linear models (LMs) treating concentration as a factor were fitted to the data using robust regression (Fig. 1a) (Huber 1981). For K=1, the linear analysis of variance (ANOVA) model assumes the form:

#(5) ymj=μm+ϵmj,

with $\mu_{m}$ denoting the mean of the mth concentration (including m=0, representing the zero concentration), j indexing replicates, and $\epsilon_{mj}$ assumed to follow a normal distribution.

When K exceeds 1, an expanded ANOVA model is fitted to the data with both concentration and time given as categorical factors, and given as follows.

#(6) ymkj=μmk+ϵmkj.

Here, $\mu_{mk}$ represents the effect of the mth concentration and kth time point. RLMs were fitted to the data through the rlm() function within the MASS R package (Huber 1981, Venables *et al.* 2002).

### 2.3 Differential expression analysis

To identify compound-induced differentially expressed genes, we compared the transcriptomic profiles of nonzero concentrations to those of the plate’s vehicle control (DMSO) (Fig. 1a). Let $\mu \left(x\right)$ represent the mean response of a gene at concentration of x. For models assuming the form of Equations (1) and (2), the statistical test encompasses the following steps:

1. Formulate null and alternative hypotheses:
  $$H_{0}:\;\mu \left(x^{*}\right)=\mu \left(0\right)$$
  $$H_{a}:\;\mu \left(x^{*}\right)\neq \mu \left(0\right)$$
2. Set the significance level: α=0.05.
3. Calculate *t*-statistics:
  #(7) t=bx*-b0β^SE,
  where SE represents the standard error of $\left[b\left(x^{*}\right)-b\left(0\right)\right]\hat{\beta }$.
4. Accept $H_{0}$ if $\left|t\right|<{\left|t\right|}_{\frac{\alpha }{2},d}$, and reject $H_{0}\;$if $\left|t\right|>{\left|t\right|}_{\frac{\alpha }{2},d}$, with d denoting the effective degrees of freedom (Wood 2017).

For statistical tests applied to other models, refer to Supplementary Methods. *P*-values were adjusted for multiple hypothesis testing via the Benjamini-Hochberg method.

### 2.4 Characterization of efficacy and potency

Each compound was categorized as a gene-specific activator or inhibitor based on the direction in which it affects the target gene’s expression. Whether to classify a compound as an activator or an inhibitor was determined by comparing the relative log_2_ fold changes of the maximum and minimum expression levels to the plate’s vehicle control (Fig. 1b). We characterized the efficacy and potency of each compound-gene pair by quantifying and estimating the standard error of the log_2_-transformed maximum change, i.e. $\log_{2}\left[E_{\max}\right]$**(**$\log_{2}\left[I_{\max}\right]$**)**, and log_10_-transformed half maximal effective (inhibitory) concentration, i.e. $\log_{10}\left[{EC}_{50}\right]$**(**$\log_{10}\left[{IC}_{50}\right]$**)**, for each GAM fitted to the data **(**see Fig. 1b for definitions of $\log_{2}\left[E_{\max}\right]$, $\log_{2}\left[I_{\max}\right]$, $\log_{10}\left[{EC}_{50}\right]$, and $\log_{10}\left[{IC}_{50}\right]$**)**. Furthermore, we developed a novel “back calculation” algorithm to estimate the standard errors of $\log_{10}\left[{EC}_{50}\right]$ and $\log_{10}\left[{IC}_{50}\right]$ efficiently. Our algorithm uses the delta method to estimate standard errors, for which we have derived a closed-form expression using implicit differentiation (Oehlert 1992), obviating the need for computationally intensive procedures like bootstrapping **(**see Supplementary Methods for the derivation of the standard error of $\log_{10}\left[{EC}_{50}\right]$ and $\log_{10}\left[{IC}_{50}\right]$**)**. It is important to note that both our method and bootstrapping are mathematically valid, but our method outperforms bootstrapping in speed by more than 800-fold (see Supplementary Methods for the comparison against bootstrapping). Note that these efficacy and potency estimates were exclusively derived from the application of GAMs, as opposed to RLMs.

## 3 Results

### 3.1 Assessment of the normality of log-transformed expression data

Both GAMs and RLMs assume that the error terms are normally distributed (see Sections 2.1 and 2.2). To justify our models, we systemically examined the normality of log-transformed gene expression data. We constructed a quantile–quantile (Q–Q) plot for the log-transformed expression data of the DMSO controls we fitted to each model. A Q–Q plot is commonly used to visually evaluate how well the distribution of the data matches a normal distribution. From the Q–Q plot, we calculated for each model the Pearson correlation coefficient ($\rho_{N}$**)**, which provides a quantitative measurement of the agreement of the normal distribution to the data (Supplementary Fig. S2a) (Filliben 1975, Vogel 1986). The resulting distribution of $\rho_{N}$ across models is heavily left-skewed, and the median value of $\rho_{N}$ reaches 0.96 (Supplementary Fig. S2b). This indicates that the assumption of normality is reasonable for the practical purpose of our analysis, justifying the adoption of t-tests.

### 3.2 Evaluating the robustness of models

To demonstrate the robustness of GAMs and RLMs, we systematically evaluated the results generated from GAMs, RLMs, and LMs using data for the same compound-cell line pairs collected from different batches. Here LMs refer to the same ANOVA models as described in Equation (5), fitted to the data using ordinary least squares regression.

By matching compounds, cell lines, time, and dose, we obtained a total of 2320 perturbation conditions tested in at least two batches, with a substantial number of concentrations (C>4). Log_2_ fold change was calculated from each model for each one of the 2 268 960 pairs of genes and replicated perturbation conditions. Standard deviations (SDs) of log_2_ fold changes and pairwise Pearson correlation coefficients ($\rho_{\mathrm{inter}}$**)** for each replicated condition were subsequently calculated between the batches.

**Figure 2.**
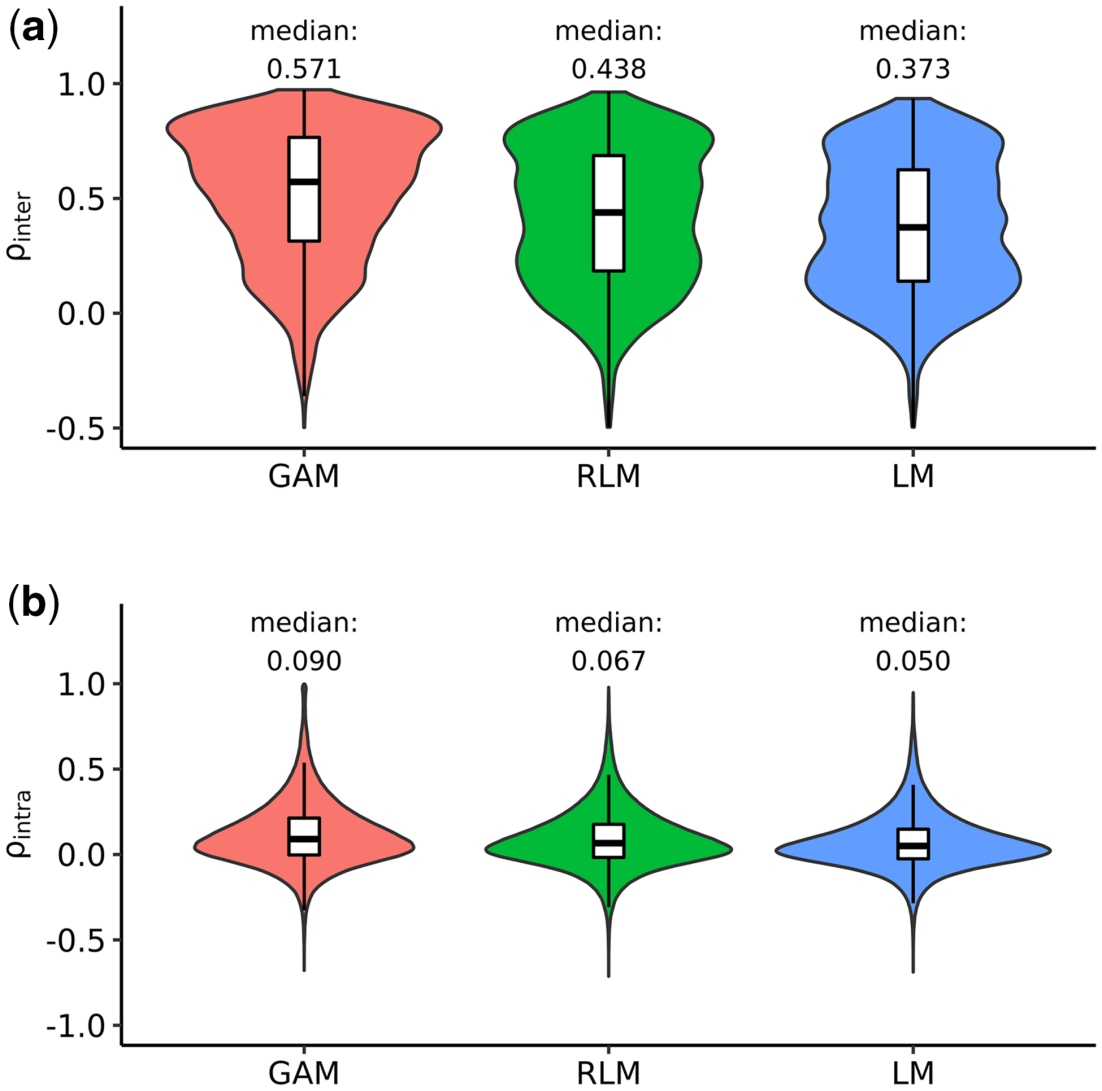
Calculation of inter- and intra-batch Pearson correlation coefficients. (a) Distribution of pairwise inter-batch Pearson correlation coefficients ($\rho_{\mathrm{inter}}$) of model-derived log_2_ fold changes for the same perturbation condition. (b) Distribution of pairwise intra-batch Pearson correlation coefficients ($\rho_{\mathrm{intra}}$) of model-derived log_2_ fold changes between perturbation conditions belonging to the same batch. GAMs, RLMs, and LMs stand for generalized additive models, robust linear models, and linear models, respectively.

The resulting distributions of SDs were more concentrated near zero for GAMs and RLMs than for LMs (Supplementary Fig. S3). The median SDs of GAMs and RLMs reached 0.169 and 0.213, representing a 32.4% and 14.8% reduction from the median SD of LMs, respectively (Supplementary Fig. S3). Consistent with SDs, the distributions of $\rho_{\mathrm{inter}}$ were more left-skewed for GAMs and RLMs compared to LMs (Fig. 2a). The median $\rho_{\mathrm{inter}}$ of GAMs and RLMs achieved 0.571 and 0.438, representing a 53.1% and 17.4% improvement over the median $\rho_{\mathrm{inter}}$ of LMs, respectively (Fig. 2a). This suggests the superior robustness of GAMs and RLMs over LMs.

### 3.3 Assessment of batch and plate effects

Another critical component of our robustness analysis is to investigate the influence of batch and plate effects on our modeling. For batch effects, we systematically compared the correlation between different batches of the same compound (i.e. inter-batch correlation) and the correlation between different compounds within the same batch (i.e. intra-batch correlation). When matching by batches, we obtained a total of approximately 1.6 million pairs of perturbation conditions belonging to the same batch. In contrast to the inter-batch correlation (Fig. 2a), the median intra-batch correlation coefficients were close to 0 for all models (Fig. 2b). This suggests that nonidentical perturbations in the same batch were, on average, lowly correlated. These findings demonstrate that batch effects had a much weaker impact on transcriptomic measurements than the actual perturbations.

To assess plate effects, we fitted generalized additive mixed models (GAMMs) and robust linear mixed models (RLMMs), treating the plate variable as a random effect across the entire dataset (see Supplementary Methods for detailed model descriptions). We compared transcriptome-wide log_2_ fold changes estimated by GAMs and RLMs with those estimated by GAMMs and RLMMs, respectively, by calculating the Pearson correlation coefficients for each perturbation condition. The resulting distribution of correlation coefficients was heavily skewed to the left, with median values exceeding 0.99 and 0.97 for GAMs and RLMs, respectively (Supplementary Fig. S4). These findings suggest that plate effects did not significantly impact our results.

### 3.4 A database of compound-induced transcriptional changes and efficacy–potency analysis

**Figure 3.**
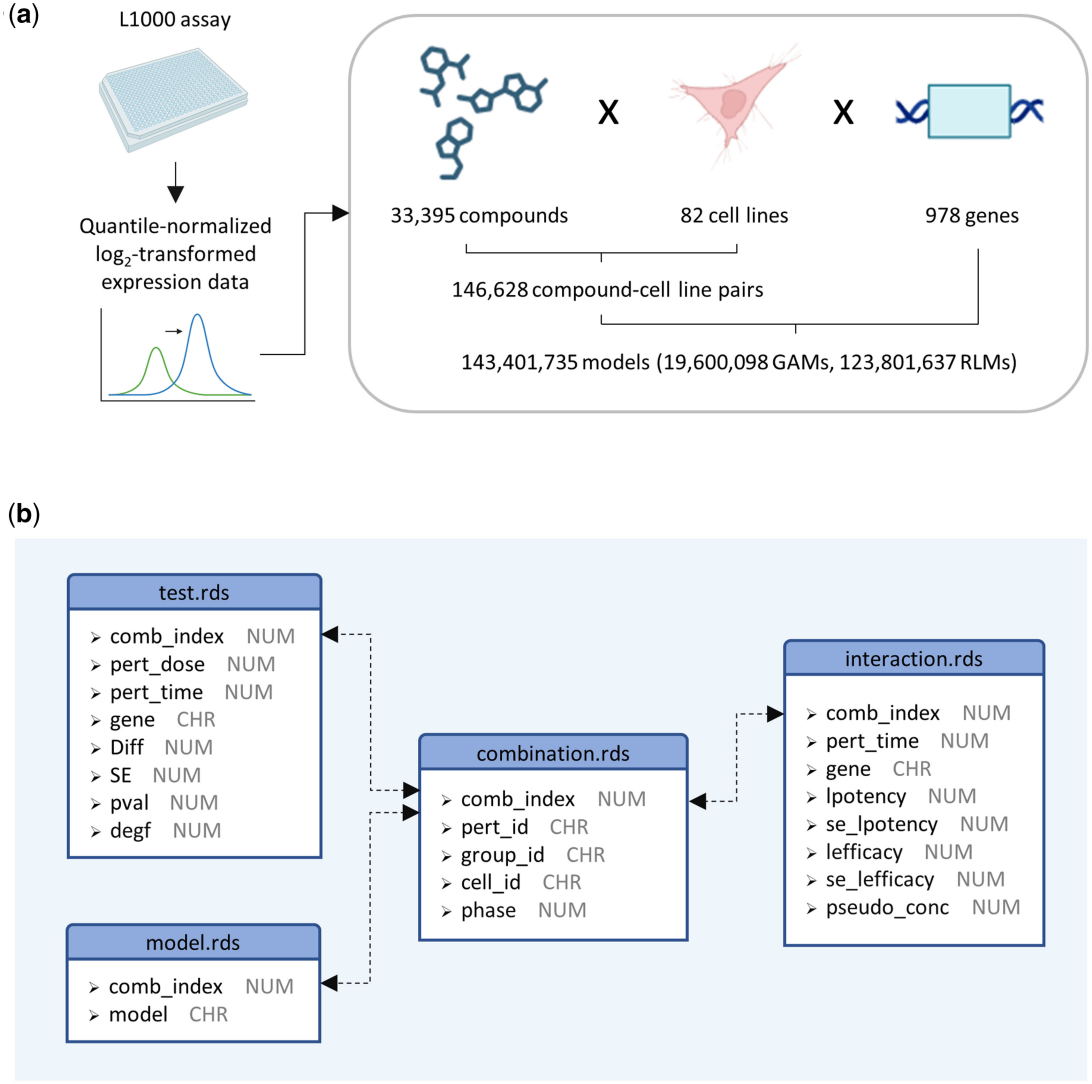
Descriptions of the DOSE-L1000 database. (a) Creation of DOSE-L1000. Quantile-normalized log_2_-transformed expression data were fitted by GAMs and RLMs, followed by differential expression analysis and potency/efficacy calculations. Over 140 million models were fitted to all unique tuples of compounds, cell lines, and genes. (b) Entity relationship diagrams illustrating the relationships between tables in the DOSE-L1000 database. Variable names and variable types are represented by the first and second words in each bullet point, respectively. NUM and CHR stand for numeric and character variables, respectively. Detailed variable descriptions can be found in Supplementary Table S3.

With the methodological foundation, we embarked on the analysis of gene expression data within the LINCS L1000 project. More than 140 million models were fitted across the vast array of compounds, cell lines, and genes present in the L1000 database (Subramanian *et al.* 2017). Our efforts encapsulated a total of 33 395 compounds, 82 cell lines, and 978 landmark genes, culminating in the assembly of the DOSE-L1000 database (Fig. 3a).

The DOSE-L1000 database is composed of four tables: combination, test, model, and interaction (Fig. 3b). These tables, meticulously structured and stored in the R Data Serialization (rds) file format, facilitate the distillation of insights from the intricate data tapestry. The model table attests to whether a given pair of compounds and cell lines was subjected to fitting using a GAM or RLM. The test table stores log_2_ fold changes and *P*-values derived from the differential expression analysis. Notably, this table extends its utility by appending standard errors of log_2_ fold changes and degrees of freedom, thus facilitating both statistical inference and the establishment of confidence intervals. The interaction table provides estimates and standard errors of $\log_{10}\left[{EC}_{50}\right]$ and $\log_{10}\left[{IC}_{50}\right]$, as well as analogous estimates and standard errors of $\log_{2}\left[E_{\max}\right]$ and $\log_{2}\left[I_{\max}\right]$ obtained through GAM fitting. Lastly, each unique pair of compounds and cell lines was assigned a unique index to enhance data organization, management, and integrity. This indexing strategy facilitated efficient querying and cross-referencing of information across the database. Note that the same compounds were occasionally tested in multiple batches (e.g. BRD-A19500257 in LJP005 and LJP006), leading to redundancy in compound records. To address this challenge, we uniquely identified each compound through a pair of BRD ID (i.e. pert_id) and batch ID (i.e. group_id) (Fig. 3b).

Data within the DOSE-L1000 database can be readily manipulated in the R programming language (R Core Team 2021). Subsequent sections will illustrate the utility of our database through a series of exemplary applications. Note that the findings unveiled in these sections are not intended to introduce novel biological insights but to demonstrate the capabilities afforded by the DOSE-L1000 database, showcasing its potential to discern, compare, and comprehend compound-induced gene expression changes across diverse biological contexts.

### 3.5 Distinctive gene expression changes unveiled by DOSE-L1000

**Figure 4.**
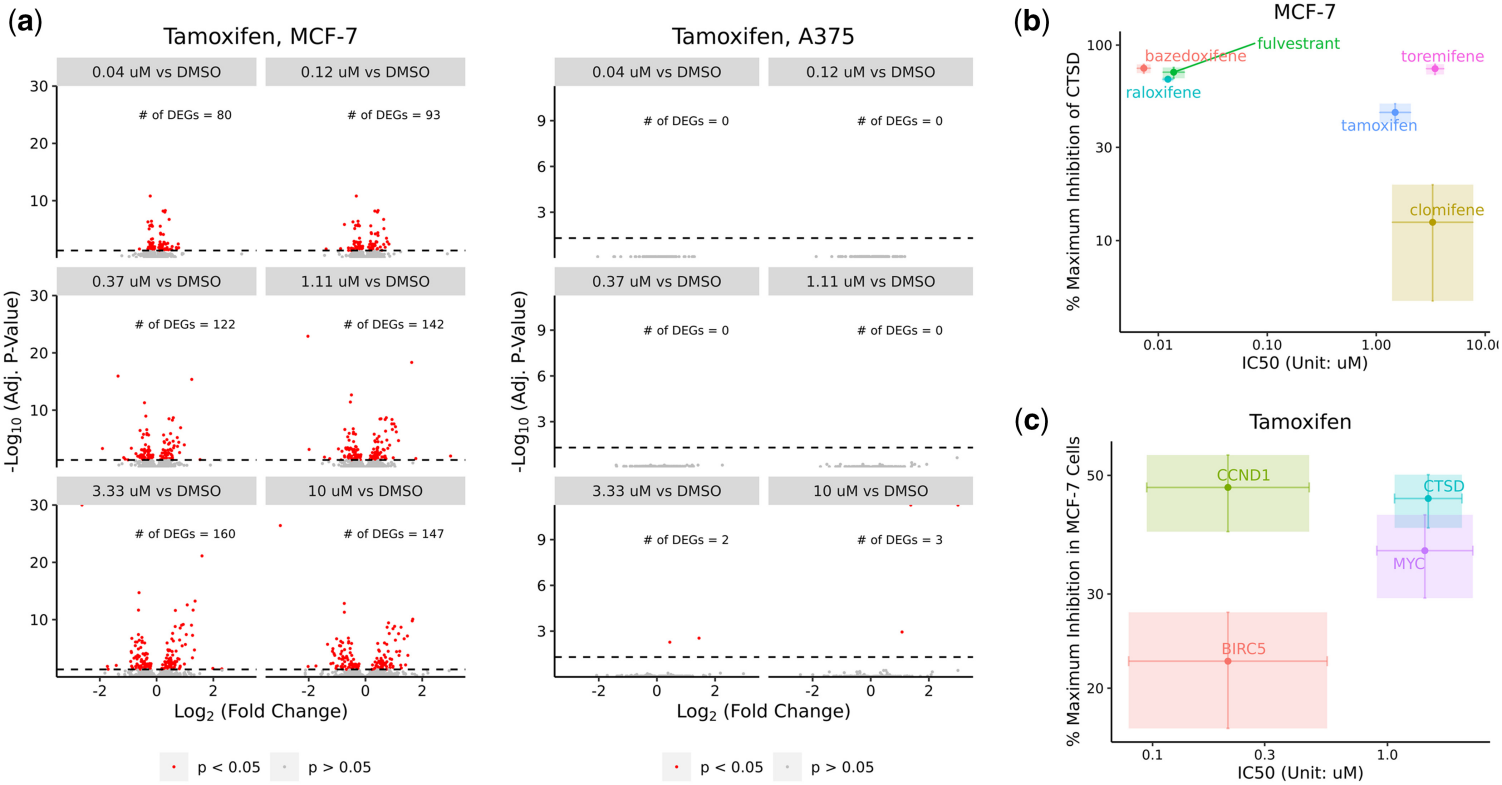
Exploring cellular responses and therapeutic interventions using DOSE-L1000. (a) Volcano plots depicting gene expression changes in MCF-7 breast cancer cells and A375 melanoma cells treated with varying concentrations of tamoxifen, based on L1000 phase 2 data (GSE70138). Dashed horizontal lines indicate an adjusted *P*-value threshold of 0.05. Differentially expressed genes (DEGs) are above dashed lines. Number of DEGs are indicated on each plot. (b) Scatter plot illustrating efficacy and potency of various SERMs and SERDs on CTSD in MCF-7 cells. ${IC}_{50}$ and % maximum inhibition of CTSD are displayed on the *x*- and *y*-axes, respectively. ${IC}_{50}$ is calculated from $10^{\log_{10}\left[{IC}_{50}\right]}-c_{p}$, where $c_{p}$ represents the pseudo concentration. % maximum inhibition of CTSD is calculated from $1-2^{\log_{2}\left[I_{\max}\right]}$. Data are shown as parameter estimates ± standard error (shaded rectangles). Both *x*- and *y*-axes are log-scaled. (c) Scatter plot illustrating tamoxifen’s efficacy and potency across different ER targets in MCF-7 cells. ${IC}_{50}$ and % maximum inhibition in MCF-7 cells are displayed on the *x*- and *y*-axes, respectively. ${IC}_{50}$ is calculated from $10^{\log_{10}\left[{IC}_{50}\right]}-c_{p}$. % maximum inhibition in MCF-7 cells is calculated from ${1-2}^{\log_{2}\left[I_{\max}\right]}$. Data are presented as parameter estimates ± standard error (shaded rectangles). Both *x*- and *y*-axes are log-scaled.

Here we explored transcriptomic variations in two cell lines, MCF-7 breast cancer cells and A375 melanoma cells, treated with tamoxifen over 24 h (Fig. 4a). Volcano plots were generated using log_2_ fold changes and *P*-values from the test table in DOSE-L1000, with *P*-values adjusted using the Benjamini Hochberg method. Tamoxifen, an estrogen receptor (ER) antagonist, induced significant transcriptomic alterations in MCF-7 cells, with a proportional increase in gene expression changes correlating to concentration (Fig. 4a). This is expected because MCF-7 cells are derived from breast tissue and are known to express ERs (Lee, Oesterreich, and Davidson 2015), making them highly responsive to hormonal interventions like tamoxifen. Conversely, tamoxifen has negligible impact on A375 cells (Fig. 4a), which is consistent with their distinct genetic and molecular makeup.

The different responses between MCF-7 and A375 cells highlight the importance of considering the specific cellular context and molecular characteristics when interpreting the effects of compounds on gene expression. This example underscores the values of DOSE-L1000 in unraveling the intricacies of compound-induced effects across diverse cellular backgrounds.

### 3.6 Compound efficacy and potency comparison enabled by DOSE-L1000

Next, we assessed the potency and efficacy of six selective estrogen receptor modulators (SERMs) and degraders (SERDs) inhibiting cathepsin D (CTSD), a recognized ER target, in MCF-7 cells (Fig. 4b). Treatment with SERMs or SERDs can modulate ER activity, leading to reduced CTSD expression as part of the broader transcriptional changes occurring in response to altered estrogen signaling (Cheng *et al.* 2012, Daniel *et al.* 2015). Leveraging DOSE-L1000, we extracted estimates of $\log_{10}\left[{IC}_{50}\right]$ and $\log_{2}\left[I_{\max}\right]$, along with their standard errors, from the interaction table. Remarkably, these ER antagonists exhibited substantial variability in both potency and efficacy, with bazedoxifene displaying both the lowest ${IC}_{50}$ and highest maximum inhibition. In contrast, tamoxifen, toremifene, and clomifene exhibited reduced potency and efficacy against CTSD (Fig. 4b). The GAMs used to fit to the data are shown in Supplementary Fig. S5.

Furthermore, we examined tamoxifen’s efficacy and potency across different ER targets (Fig. 4c). The GAMs used for data fitting are shown in Supplementary Fig. S6. Among the evaluated genes, cyclin D1 (CCND1) emerged as the most responsive in MCF-7 cells, characterized by an ${IC}_{50}$ of 0.21 uM and maximum inhibition of 47% (Fig. 4c). On the other hand, MYC and CTSD exhibited higher ${IC}_{50}$ values compared to CCND1, suggesting that a greater concentration of tamoxifen is required for equally effective inhibition of MYC and CTSD expression (Fig. 4c). This approach, which can be readily applied to other compound-gene pairs, serves as a valuable tool for scrutinizing target engagement and off-target effects of diverse compounds.

### 3.7 Clustering of small molecules enabled by DOSE-L1000

**Figure 5.**
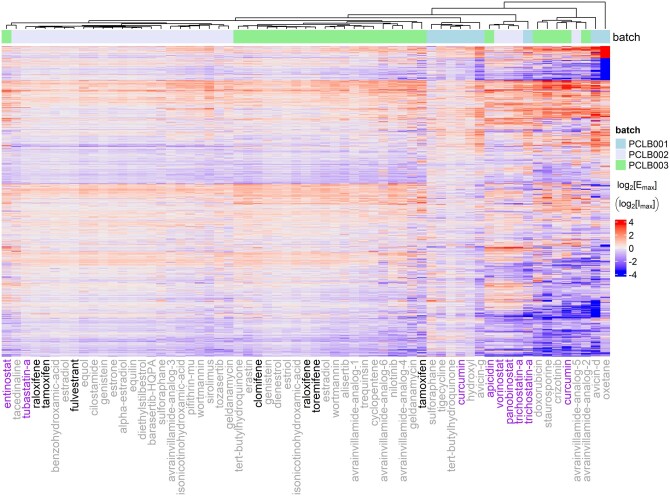
Heat map revealing the spectrum of efficacy of compound-gene pairs in HepG2 cells across the transcriptome. Constructed using L1000 phase 1 data (GSE92742), each row corresponds to a gene, while each column corresponds to a compound, with compound names indicated below the heat map. SERMs, SERDs, and HDAC inhibitors are highlighted. Only compounds with valid generic names are included. The color of the voxel represents log_2_-transformed maximum effect or inhibition of the respective compound-gene pair. Batch information is indicated by color above the heat map. Dendrograms atop represent hierarchical clusters generated through the Ward algorithm based on Euclidean distance.

Our utilization of DOSE-L1000 extended to clustering analysis, a technique commonly used to unveil patterns within complex data. Specifically, we applied hierarchical clustering to examine the transcriptome-wide efficacy profiles of small molecules tested in HepG2 cells, a widely used cell line model for hepatotoxicity investigations, during the first phase of the L1000 project (Fig. 5) (Ward 1963, Gu *et al.* 2016). Among these molecules were SERMs and SERDs—tamoxifen, raloxifene, clomifene, toremifene, and fulvestrant—which induced relatively modest transcriptional changes in HepG2 cells (Fig. 5). In contrast, a cohort of histone deacetylase (HDAC) inhibitors, including curcumin, apicidin, vorinostat, panobinostat, and Trichostatin A, elicited more pronounced changes, indicating a potentially heightened transcriptomic response of hepatocytes to HDAC inhibition (Fig. 5).

Given our hypothesis that early changes in gene expression upon drug treatment could provide insights into later physiological events, we postulated that the observed transcriptome-wide efficacy profiles might offer clues regarding compound mechanisms of action or toxicity. Intriguingly, within the spectrum of HDAC inhibitors, entinostat and Tubastatin A stood out for inducing relatively mild transcriptomic changes in HepG2 cells. This analysis showcases the potential of DOSE-L1000 to unveil nuances in compounds belonging to the same class, thereby contributing to a deeper understanding of drug effects and mechanisms.

### 3.8 Predictions of drug–target interactions based on expression similarity

The vast amount of data in DOSE-L1000 also opens up opportunities for investigating drug mechanisms. Given that drugs with similar mechanisms of action (MOAs) generally lead to similar gene expression changes, we sought to investigate how accurately gene expression similarities could inform known protein targets of drugs. Specifically, we focused on compounds targeting HDACs across three cancer cell lines: MCF-7 (breast), PC-3 (prostate), and A549 (lung) cancer cells.

**Figure 6.**
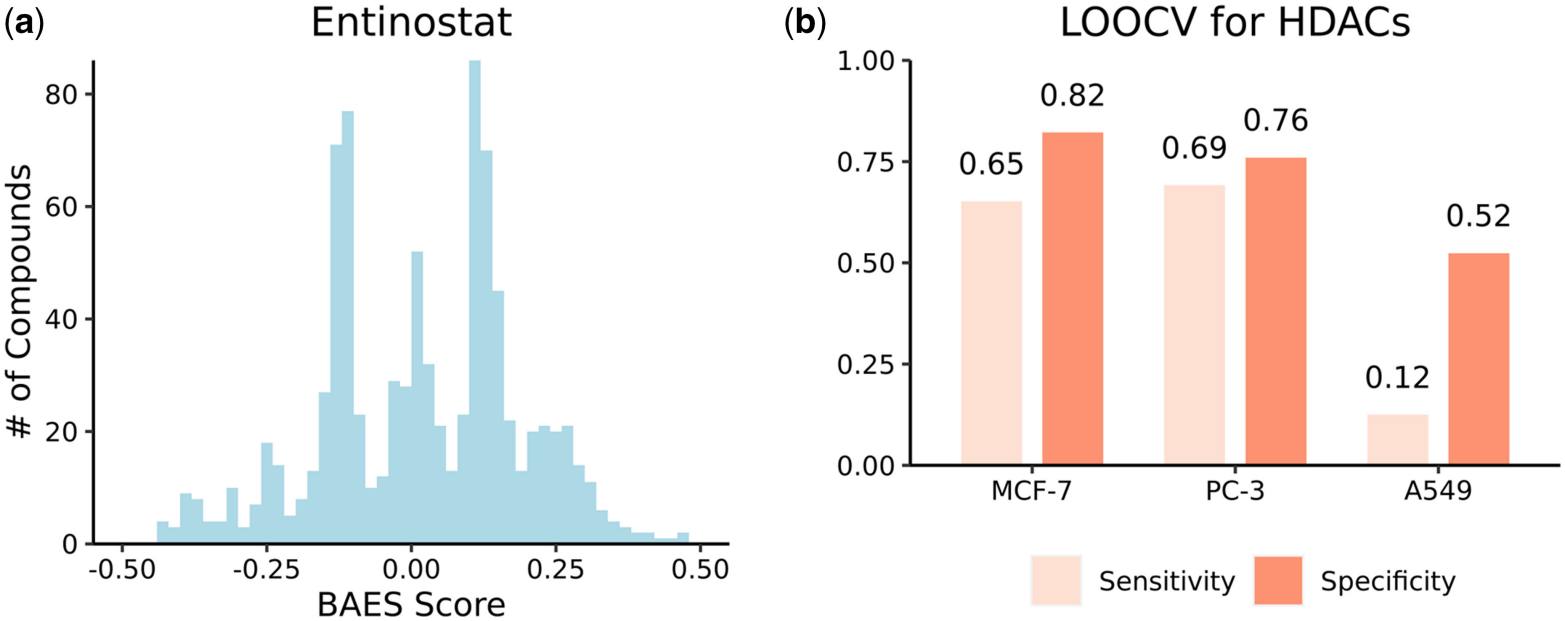
Classification of HDAC inhibitors. (a) Frequency distribution of the Bridge Adjusted Expression Similarity (BAES) scores of DOSE-L1000 compounds compared to entinostat, an HDAC inhibitor. (b) Sensitivity and specificity of the HDAC inhibitor classifiers on validation datasets across cell lines, obtained through leave-one-out cross-validation (LOOCV).

We initiated this process by identifying a comprehensive list of compounds known to target HDACs from Drug Target Commons (Tang *et al.* 2018). By following a previously described method, we then computed the Bridge Adjusted Expression Similarity (BAES) scores of DOSE-L1000 compounds compared to the known HDAC inhibitors (Fig. 6a) (Wang *et al.* 2013). Subsequently, a binary classifier was developed to distinguish between compounds that do and do not target HDACs based on the BAES scores in each cell line (Wang *et al.* 2013). The performance of the classifier was evaluated using leave-one-out cross-validation (LOOCV) (see Supplementary Methods for details) (Wang *et al.* 2013).

Among the three classifiers, the models for MCF-7 cells and PC-3 cells demonstrated similar performance, achieving a balanced accuracy of 0.74 and 0.73, respectively (Fig. 6b). This suggests a cell line-specific positive association between expression similarity and drug–target interactions. In contrast, the same modeling approach did not translate well to A549 cells (Fig. 6b). Compared to MCF-7 and PC-3 cells, the unique molecular characteristics of A549 cells may have contributed to a more diverse landscape of transcriptional response to different HDAC inhibitors.

Furthermore, ranking by the BAES scores, we observed surprisingly high values for pravastatin, an HMG-CoA reductase inhibitor, in MCF-7 cells. Although pravastatin is widely used to treat hypercholesterolemia, several other statin molecules have previously demonstrated anti-cancer effects via HDAC inhibition (Vigushin *et al.* 2001, Shen *et al.* 2020). Our findings suggest a potential inhibitory role of pravastatin on HDACs, an MoA not captured by its known targets (Haggag *et al.* 2021).

## 4 Discussion

Our study presents a robust model-based framework that sheds light on intricate compound-induced transcriptional changes within the LINCS L1000 data. Through the application of over 140 million GAMs and RLMs, we systematically explored the interplay between compounds, genes, and cell lines encoded in the L1000 database. This framework enabled the evaluation of potency and efficacy of approximately 20 million compound-gene pairs, uncovering a wealth of insights.

To assess model robustness, we conducted a systematic comparison of GAMs, RLMs, and LMs for replicated perturbation conditions. The advantage of RLMs over LMs can be explained by the ability of RLMs to downweight the influence of outliers. However, in both LMs and RLMs, dose is treated as a categorical variable, and a distinct response value is fitted to each dose level without regard to its order. Hence, RLMs may still break down when data variability for a particular dose is inherently large. In contrast, despite requiring a substantial number of doses (e.g. C>4), GAMs confer improved robustness by treating dose as a continuous variable and using “nearby” concentrations to constrain expression level estimation from overshooting (Supplementary Figs S5 and S6). Furthermore, GAMs facilitate the estimation of efficacy and potency **(**e.g. $\log_{10}\left[{IC}_{50}\right]$**)**, whereas RLMs do not. The superior performance of GAMs highlights the practical benefits of this model in the analysis of dose–response data.

Furthermore, we systematically examined batch and plate effects to justify our approach. Although moderated t-tests (i.e. LIMMA) can be extended to RLMs and GAMs (Ritchie *et al.* 2015), as LIMMA only affects the estimation of standard errors without altering coefficients themselves, we do not anticipate it providing substantial additional insights beyond what is already offered by our framework.

A major contribution of our work lies in the creation of the DOSE-L1000 database. This database comprises four meticulously structured tables stored in the rds format, offering a well-organized collection of information. Notably, these rds-formatted tables can be readily transformed into an SQL database, enhancing the scalability and interoperability of the data. The transition to an SQL database offers several advantages, including efficient data storage and the ability to execute complex queries. To ensure the sustainability of our database, we will explore the implementation of an automated update mechanism to streamline the data ingestion process.

Another noteworthy contribution is the derivation of the closed-form expression of the standard errors of $\log_{10}\left[{EC}_{50}\right]$ and $\log_{10}\left[{IC}_{50}\right]$. This enables the delta method to efficiently estimate the standard errors of potency, surpassing bootstrapping (R=1000) by more than 800-fold in speed (Oehlert 1992). In addition to dose–response data, our algorithm can also be used to calculate standard errors of time-response data (Wang and Belta 2019, Wang *et al.* 2020).

DOSE-L1000 offers a wealth of possibilities for diverse applications across the scientific community. As shown in Sections 3.5–3.8, DOSE-L1000 enables a deep exploration of target gene expression changes in response to chemical perturbations and the cell-line specificity of such changes. Furthermore, it holds the potential to fuel predictive models that anticipate drug–target interactions based on gene expression similarity (Iorio *et al.* 2010, Iskar *et al.* 2010, Wang *et al.* 2016, van Hasselt *et al.* 2020, Zhu *et al.* 2021). Using a more sophisticated model could probably further enhance the classification performance. However, it is important to note that relying solely on overall expression similarity might not always lead to accurate predictions of drug MoAs. We have observed instances where cancer cells exhibit largely similar transcriptomic responses to different anti-cancer agents due to shared pathways like apoptosis. This underscores the importance of selecting genes that most accurately represent the unique MoAs of each therapeutic agent.

For enhanced data access, institutions with the necessary resources may further consider augmenting DOSE-L1000 with a user-friendly front-end web interface. Such an interface would empower researchers and stakeholders to effortlessly query and visualize the extensive dataset, thus fostering collaborative and interdisciplinary investigations.

While our approach offers valuable insights, it is essential to acknowledge its limitations. For example, GAMs are unable to extrapolate outside the dose ranges. Furthermore, our analysis assumes normally distributed errors in the GAMs, facilitating straightforward statistical inference. However, GAM modeling can accommodate other error structures or data distributions. For example, errors can be assumed to follow a scaled t-distribution, which could potentially further enhance model robustness (Wood 2016). It is important to note that a change in the error structure may lead to computational challenges. Specifically, the formula for calculating the test statistic under scaled *t*-distributed errors might not have a closed-form expression, requiring more complex numeric methods for hypothesis testing and confidence interval estimation.

In conclusion, DOSE-L1000 serves as a groundbreaking resource for unveiling the intricate landscape of compound-induced transcriptional changes and also paves the way for a diverse spectrum of applications. Through its insights and versatility, DOSE-L1000 shapes a new paradigm for leveraging the wealth of LINCS L1000 data, empowering researchers across domains to accelerate the trend toward omics-driven drug discovery.

## Supplementary Material

btad683_Supplementary_Data

## Data Availability

The DOSE-L1000 database is available at https://doi.org/10.5281/zenodo.8286375. The original LINCS L1000 Level 3 data have been previously published and can be accessed at GEO with dataset identifiers GSE92742 (https://www.ncbi.nlm.nih.gov/geo/query/acc.cgi?acc=GSE92742) and GSE70138 (https://www.ncbi.nlm.nih.gov/geo/query/acc.cgi?acc=GSE70138) (Subramanian *et al.* 2017). All code is available at https://github.com/JmWangBio/DoseL1000.
