# Supplementary material for: DOSE-L1000: unveiling the intricate landscape of compound-induced transcriptional changes
Source: Bioinformatics. 2023 Nov 11;39(11):btad683. doi: 10.1093/bioinformatics/btad683 (PMC10663987; doi:10.1093/bioinformatics/btad683)
Supplement: btad683_Supplementary_Data [file btad683_supplementary_data.pdf]

DOSE-L1000: Unveiling the Intricate Landscape of Compound-Induced Transcriptional Changes

Junmin Wang[1,#,*], **Steven Novick**[2,#]

[1] *Data Sciences and Quantitative Biology, Discovery Sciences, Biopharmaceuticals R&D, AstraZeneca, Gaithersburg, Maryland*

[2] *Global Statistical Sciences, Eli Lilly, Indianapolis, Indiana*

[#]*These authors contributed equally to this work.*

[*]*Corresponding author: Junmin Wang (junmin.wang@astrazeneca.com)*

Supplementary Information

- Supplementary Methods
- Supplementary Tables
- Supplementary Figures
- Supplementary References

Supplemental Methods

Steps of the statistical tests for identifying differentially expressed genes

Let $\mu(x,t)$ represent the mean response of a gene at concentration of x and time of t. For models assuming the form of Eq. (3) and Eq. (4) in the main text, the statistical test entails the following steps:

1. State null and alternative hypotheses:

$$H_0: \mu(x^*, t^*) = \mu(0, t^*)$$

$$H_a: \mu(x^*, t^*) \neq \mu(0, t^*)$$

2. Set the significance level: $\alpha = 0.05$.
3. Calculate t-statistics:

$$t = \frac{[b(x^*) - b(0)]\hat{\beta}_{t^*}}{SE},$$

   where SE represents the standard error of $[b(x^*) - b(0)]\hat{\beta}_{t^*}$.

4. Accept H_0 if $|t| < |t|_{\frac{\alpha}{2},d}$, and reject H_0 if $|t| > |t|_{\frac{\alpha}{2},d}$ with d denoting the effective degrees of freedom.

Let $\mu(x)$ represent the mean response of a gene at concentration of x. For models assuming the form of Eq. (5) in the main text, the statistical test entails the following steps:

1. State null and alternative hypotheses:

$$H_0: \mu(x^*) = \mu(0)$$

$$H_a: \mu(x^*) \neq \mu(0)$$

2. Set the significance level: $\alpha = 0.05$.
3. Calculate t-statistics:

$$t = \frac{\hat{\mu}_{x^*} - \hat{\mu}_0}{SE},$$

   where SE represents the standard error of $(\hat{\mu}_{x^*} - \hat{\mu}_0)$.

4. Accept H_0 if $|t| < |t|_{\frac{\alpha}{2},N-P}$, and reject H_0 if $|t| > |t|_{\frac{\alpha}{2},N-P}$, where N and P denote the number of observations and the number of parameter estimates, respectively.

Let $\mu(x,t)$ represent the mean response of a gene at concentration of x and time of t. For models assuming the form of Eq. (6) in the main text, the statistical test entails the following steps:

1. State null and alternative hypotheses:

$$H_0: \mu(x^*, t^*) = \mu(0, t^*)$$

$$H_a: \mu(x^*, t^*) \neq \mu(0, t^*)$$

2. Set the significance level: $\alpha = 0.05$.
3. Calculate t-statistics:

$$t = \frac{\hat{\mu}_{x^*t^*} - \hat{\mu}_{0t^*}}{SE},$$

where SE represents the standard error of $(\hat{\mu}_{x^*t^*} - \hat{\mu}_{0t^*})$.

4. Accept H_0 if $|t| < |t|_{\frac{\alpha}{2},N-P}$, and reject H_0 if $|t| > |t|_{\frac{\alpha}{2},N-P}$, where N and P denote the number of observations and the number of parameter estimates, respectively.

Back calculation algorithm

Consider the GAM described by Eq. (1) and Eq. (2) in the main text:

$$y_i = f_\beta(x_i) + \epsilon_i,$$

$$f_\beta(x_i) = b(x_i)\beta.$$

Let's assume, without loss of generality, that the compound inhibits the target gene, leading to y_{min} achieved at x^*. It is easy to show that the standard error of the estimated $\log_2[I_{max}]$, i.e., $y_{min} - y_0$, is given by:

$$\{[b(x^*) - b(0)]Var(\hat{\beta})[b(x^*) - b(0)]^T\}^{\frac{1}{2}}.$$

Let x^{50} (i.e., $\log_{10}[IC_{50}]$) denote the log$_{10}$ concentration at which y achieves the midpoint between y_{min} and y_0, i.e., $y^{50} = \frac{y_{min}+y_0}{2}$. We can show that if $f_\beta{}'(x^{50})$ is non-zero, the standard error of the estimated x^{50} is given by:

$$\left\{\begin{bmatrix} -\frac{b_1(x^{50})}{\sum_{j=1}^{J}\beta_j\frac{\partial b_j(x^{50})}{\partial x}} & \cdots & -\frac{b_J(x^{50})}{\sum_{j=1}^{J}\beta_j\frac{\partial b_j(x^{50})}{\partial x}} \end{bmatrix} Var(\hat{\beta}) \begin{bmatrix} -\frac{b_1(x^{50})}{\sum_{j=1}^{J}\beta_j\frac{\partial b_j(x^{50})}{\partial x}} & \cdots & -\frac{b_J(x^{50})}{\sum_{j=1}^{J}\beta_j\frac{\partial b_j(x^{50})}{\partial x}} \end{bmatrix}^T\right\}^{\frac{1}{2}}.$$

Proof:

First, let's consider the case where $f_\beta{}'(x^{50})$ is non-zero. It is imperative that $f_\beta{}'(x^{50})$ is negative; otherwise, x^{50} would not be the minimal log$_{10}$-transformed concentration where y achieves the midpoint between y_0 and y_{min}. This prerequisite enables us to establish a positive δ such that f_β is monotonically decreasing and hence invertible on the interval $(x^{50} - \delta, x^{50} + \delta)$.

Let f be the function such that $f(\beta, x^a) = f_\beta(x^a)$, and g be the function such that $g\left(\beta, f_\beta(x^a)\right) = x^a$ for all x^a in $(x^{50} - \delta, x^{50} + \delta)$. This implies $f\big(\beta, g(\beta, y^a)\big) = y^a$ for all y^a in $\left(f_\beta(x^{50} + \delta), f_\beta(x^{50} - \delta)\right)$.

Taking the partial derivative of f with respect to $\beta_{j^*} (1 \le j^* \le J)$ at x^{50}:

$$\frac{\partial f\left(\beta, g(\beta, y^{50})\right)}{\partial \beta_{j^*}} = \sum_{j=1}^{J} \beta_j \frac{\partial b_j(x^{50})}{\partial x} \frac{\partial g(\beta, y^{50})}{\partial \beta_{j^*}} + b_{j^*}(x^{50}) = 0.$$

This implies:

$$\frac{\partial g(\beta, y^{50})}{\partial \beta_{j^*}} = -\frac{b_{j^*}(x^{50})}{\sum_{j=1}^{J} \beta_j \frac{\partial b_j(x^{50})}{\partial x}}.$$

Therefore, the standard error of the estimated x^{50}, i.e., $\log_{10}[IC_{50}]$, is given by:

$$\left\{\frac{\partial g(\beta, y^{50})}{\partial \beta} Var(\hat{\beta}) \frac{\partial g(\beta, y^{50})}{\partial \beta}^T\right\}^{\frac{1}{2}},$$

where

$$\frac{\partial g(\beta, y^{50})}{\partial \beta} = \left[\frac{\partial g(\beta, y^{50})}{\partial \beta_1} \quad \cdots \quad \frac{\partial g(\beta, y^{50})}{\partial \beta_J}\right] = \left[-\frac{b_1(x^{50})}{\sum_{j=1}^{J} \beta_j \frac{\partial b_j(x^{50})}{\partial x}} \quad \cdots \quad -\frac{b_J(x^{50})}{\sum_{j=1}^{J} \beta_j \frac{\partial b_j(x^{50})}{\partial x}}\right].$$

In cases where $f_\beta{}'(x^{50})$ equals zero, we resort to bootstrap resampling to calculate the standard error of x^{50}. Similar solutions can be derived for GAMs described by Eq. (3) and Eq. (4) in the main text.

Comparison between the delta method and bootstrapping

Bootstrapping is a common method to estimate standard errors when closed-form solutions do not exist. It typically involves at least 1000 resamples to ensure reliable results, making it a time-consuming process. Deriving the closed-form expression (as detailed in the previous section) enables the delta method to efficiently calculate the standard error of potency. To understand the advantage of the delta method in speed, we compared its performance against bootstrapping using simulated data. Data (i.e., ground truth) were simulated based on the following equation: $y = 5 + \frac{10^x}{10^x+1} + \epsilon$, where x and y denote the $\log_{10}$-transformed compound concentration and $\log_2$-transformed gene expression level, respectively. x ranges from -3 to 3 in increments of 0.5. ϵ is the normally distributed error term with mean equal to 0 and standard deviation equal to 0.1, i.e., $\epsilon \sim N(0, 0.1)$. We applied the delta method and bootstrapping with 1000 resamples separately to estimate the standard errors of $\log_2[E_{max}]$ and $\log_{10}[EC_{50}]$. The delta method ran 813 times faster than bootstrapping. Our findings can be generalized to real-world settings, as the data we simulated here have the same structure as the gene expression data we fitted the models to. Given the need to fit nearly 20 million GAMs to the data, we determined that the delta method enabled by the closed-form expression of the standard error was the key to efficiently accomplishing this task.

Generalized additive mixed models and robust linear mixed models

In cases where the number of time points equals 1, generalized additive mixed models (GAMMs) treating the plate variable as a random effect takes the form:

$$y_{iu} = f_\beta(x_{iu}) + \gamma_u + \epsilon_{iu}$$

Here, y_{iu} represents the $\log_2$-transformed gene expression level in the ith sample on the uth plate, x_{iu} is the $\log_{10}$-transformed compound concentration, γ_u is the plate effect, and ϵ_{iu} is the normally distributed error term. The smoothing spline function f_β is defined the same as in Eq. (2) in the main text. Model fitting is implemented using the gam() function within the mgcv R package (Wood 2017).

Under the same circumstances, robust linear mixed models (RLMMs) treating the plate variable as a random effect takes the form:

$$y_{mvj} = \mu_m + z_v + \epsilon_{mvj}$$

Here, μ_m denotes the mean of the mth concentration (including $m = 0$, representing the zero concentration), z_v denotes the plate effect, j indexes replicates, and ϵ_{mvj} is assumed to follow a normal distribution. Model fitting is implemented using the rlmer() function within the robustlmm R package (Koller 2016).

For cases where the number of time points exceeds 1, GAMMs and RLMMs can be derived similarly as explained in Sections 2.1 and 2.2 in the main text.

Development and cross-validation of the HDAC inhibitor classifier

To identify known HDAC inhibitors, we obtained the complete compound-target interaction dataset from Drug Target Commons (Tang et al. 2018). A compound was considered as an HDAC inhibitor if its gene symbol and activity type matched the keywords "HDAC" and "INHIBITION", respectively, in more than five instances. The efficacy profiles of all DOSE-L1000 compound-gene pairs available within the MCF-7, PC-3, and A549 cells at 6 hours were utilized for subsequent analysis. HDAC inhibitors were designated as benchmark ligands, while the remaining compounds constituted the background drugs.

Bridge Adjusted Expression Similarity (BAES) scores, as previously described, were computed (Wang et al. 2013). For enhanced clarity, we outline the detailed steps of this calculation here. Genes were ranked by $\log_2$ fold change, with the most upregulated genes at the top and the most downregulated genes at the bottom. We employed the gene set enrichment analysis (GSEA) algorithm to compare the efficacy profile of one compound (the query set) to another (the reference set) (Subramanian et al. 2005). For any given compound, all 978 landmark genes were included in the query set. Subsequently, a different compound from the list of HDAC inhibitors was chosen as the reference. All differentially expressed genes of the reference compound, defined as those with adjusted p-values less than 0.05, were included in the reference set. The GSEA algorithm generated an enrichment score, reflecting the degree of similarity between the queried compound and the reference compound (Subramanian et al. 2005). A pair of drugs could yield two enrichment scores by interchanging the roles of the query set and reference set. The BAES score was computed as the average of these two enrichment scores. The GSEA algorithm was implemented using the fgsea() function in the fgsea R package (Korotkevich et al. 2016).

To identify known gene-target pairs, we calculated the likelihood of interaction (LOI) as the average BAES score to the benchmark ligands and developed a simple binary classifier: compounds with LOI exceeding the threshold were classified as HDAC inhibitors, while those falling below the LOI threshold were not (Wang et al. 2013). The optimal LOI threshold was determined by selecting the threshold that maximized balanced accuracy. The performance of the classifier was evaluated using leave-one-out cross-validation (LOOCV). Each DOSE-L1000 compound, either a benchmark ligand or a background drug, took turns as the validation set. The remaining compounds constituted the training set. The class of the compound in the validation set was predicted based on the threshold determined from the training set. The sensitivity and specificity of the classifier were evaluated based on all validation sets combined.

Supplementary Tables

GEO Accession Code	Project Phase	URL	Files
GSE92742	1	https://www.ncbi.nlm.nih.gov/geo/query/acc.cgi?acc=GSE92742	• GSE92742_Broad_LINCS_Level3_INF_mlr12k_n1319138x12328.gctx.gz<br>• GSE92742_Broad_LINCS_gene_info.txt.gz<br>• GSE92742_Broad_LINCS_inst_info.txt.gz<br>• GSE92742_Broad_LINCS_pert_info.txt.gz
GSE70138	2	https://www.ncbi.nlm.nih.gov/geo/query/acc.cgi?acc=GSE70138	• GSE70138_Broad_LINCS_Level3_INF_mlr12k_n345976x12328_2017-03-06.gctx.gz<br>• GSE70138_Broad_LINCS_gene_info_2017-03-06.txt.gz<br>• GSE70138_Broad_LINCS_inst_info_2017-03-06.txt.gz<br>• GSE70138_Broad_LINCS_pert_info_2017-03-06.txt.gz

Supplementary Table 1. Complete list of files downloaded from the LINCS L1000 project via Gene Expression Omnibus (Subramanian et al. 2017; Edgar, Domrachev, and Lash 2002).

Concentration (uM)	Pseudo-concentration	Log_{10} (Concentration)
0 (DMSO)	$0.04 \times \left(\frac{0.04}{0.12}\right)^2$	$\log_{10}\left[0 + 0.04 \times \left(\frac{0.04}{0.12}\right)^2\right]$
0.04		$\log_{10}\left[0.04 + 0.04 \times \left(\frac{0.04}{0.12}\right)^2\right]$
0.12		$\log_{10}\left[0.12 + 0.04 \times \left(\frac{0.04}{0.12}\right)^2\right]$
0.37		$\log_{10}\left[0.37 + 0.04 \times \left(\frac{0.04}{0.12}\right)^2\right]$
1.11		$\log_{10}\left[1.11 + 0.04 \times \left(\frac{0.04}{0.12}\right)^2\right]$
3.33		$\log_{10}\left[3.33 + 0.04 \times \left(\frac{0.04}{0.12}\right)^2\right]$
10		$\log_{10}\left[10 + 0.04 \times \left(\frac{0.04}{0.12}\right)^2\right]$

Supplementary Table 2. An example illustrating how a pseudo-concentration is added to the baseline to address the challenge of log-transforming zero values. Pseudo-concentration is set equal to the lowest non-zero concentration times the squared ratio of the lowest non-zero concentration to the second lowest non-zero concentration. Log_{10} (Concentration) is calculated as the $\log_{10}$-transformed sum of baseline and pseudo-concentration.

Table Name	Variable Name	Variable Type	Description
combination	comb_index	numeric	a unique identifier for a combination of compounds, batches, and cell lines
	pert_id	character	a unique identifier for a compound, not to any particular batch
	group_id	character	a unique identifier for a batch
	cell_id	character	a CMap identifier number assigned to each cell line used in the L1000 assay
	phase	numeric	phase of the L1000 project; phase 1 and phase 2 culminate in GSE92742 and GSE70138, respectively.
model	comb_index	numeric	same as “comb_index” in “combination”
	model	character	type of the model: generalized additive model (GAM) or robust linear model (RLM)
test	comb_index	numeric	same as “comb_index” in “combination”
	pert_dose	numeric	amount of compound used to treat cells (unit: uM)
	pert_time	numeric	length of time, expressed as a number, that a compound was applied to the cells (unit: hr)
	gene	character	Entrez gene ID
	Diff	numeric	$\log_2$ fold change
	SE	numeric	standard error of $\log_2$ fold change
	pval	numeric	p-value reported from the t-test
	degf	numeric	(effective) degrees of freedom
interaction	comb_index	numeric	same as “comb_index” in “combination”
	pert_time	numeric	same as “pert_time” in “test”
	gene	character	same as “gene” in “test”
	lpotency	numeric	estimate of $\log_{10}[EC_{50}]$ or $\log_{10}[IC_{50}]$
	se_lpotency	numeric	standard error of estimated $\log_{10}[EC_{50}]$ or $\log_{10}[IC_{50}]$
	lefficacy	numeric	estimate of $\log_2[E_{max}]$ or $\log_2[I_{max}]$
	se_lefficacy	numeric	standard error of estimated $\log_2[E_{max}]$ or $\log_2[I_{max}]$
	pseudo_conc	numeric	pseudo concentration (unit: uM)

Supplementary Table 3. Complete list of tables and variables in the DOSE-L1000 database (Subramanian et al. 2017).

Supplementary Figures

Phase 1 (GSE92742)

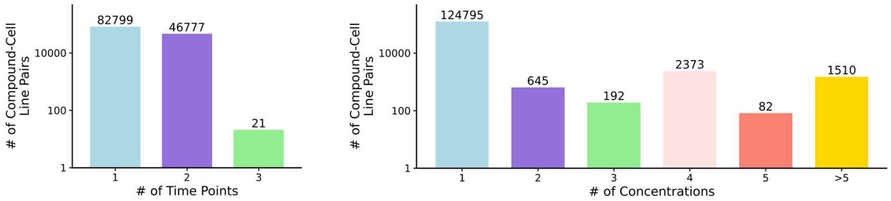

Phase 2 (GSE70138)

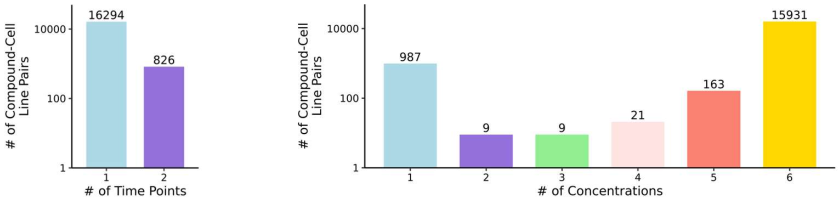

Supplementary Fig. 1. Histograms illustrating the frequency of the number of time points and concentrations (DMSO excluded) within LINCS L1000 data.

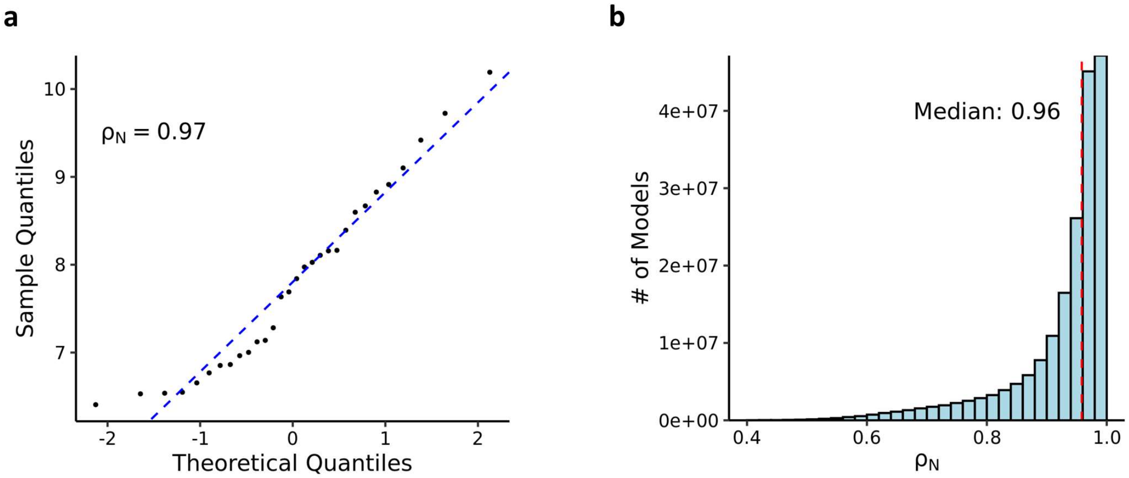

Supplementary Fig. 2. Assessment of the assumption of normality. **a** Calculation of Pearson correlation coefficients (ρ_N) from the Q-Q plot. **b** Frequency distribution of ρ_N estimated from the Q-Q plots across models.

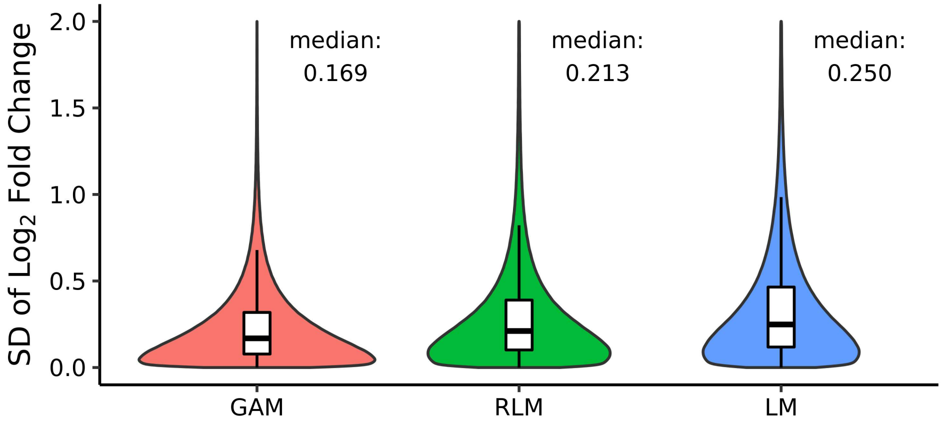

Supplementary Fig. 3. Violin plots illustrating the distribution of standard deviation (SD) of model-derived $\log_2$ fold changes between batches. GAMs, RLMs, and LMs stand for generalized additive models, robust linear models, and linear models, respectively.

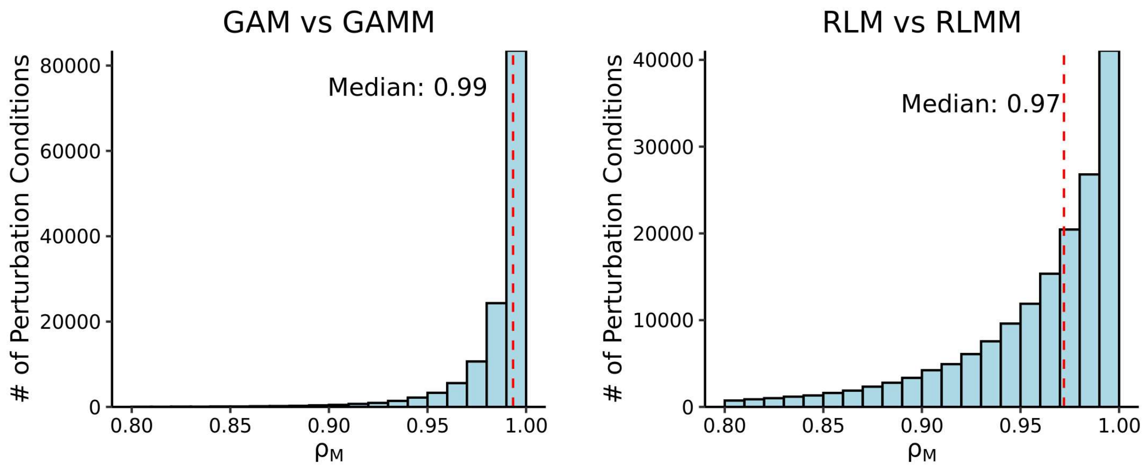

Supplementary Fig. 4. Histograms illustrating the frequency of Pearson correlation coefficients (ρ_M) of transcriptome-wide $\log_2$ fold changes of gene expression estimated by generalized additive models (GAMs) and robust linear models (RLMs) compared to their mixed-model counterparts (GAMMs and RLMMs), respectively for each perturbation condition.

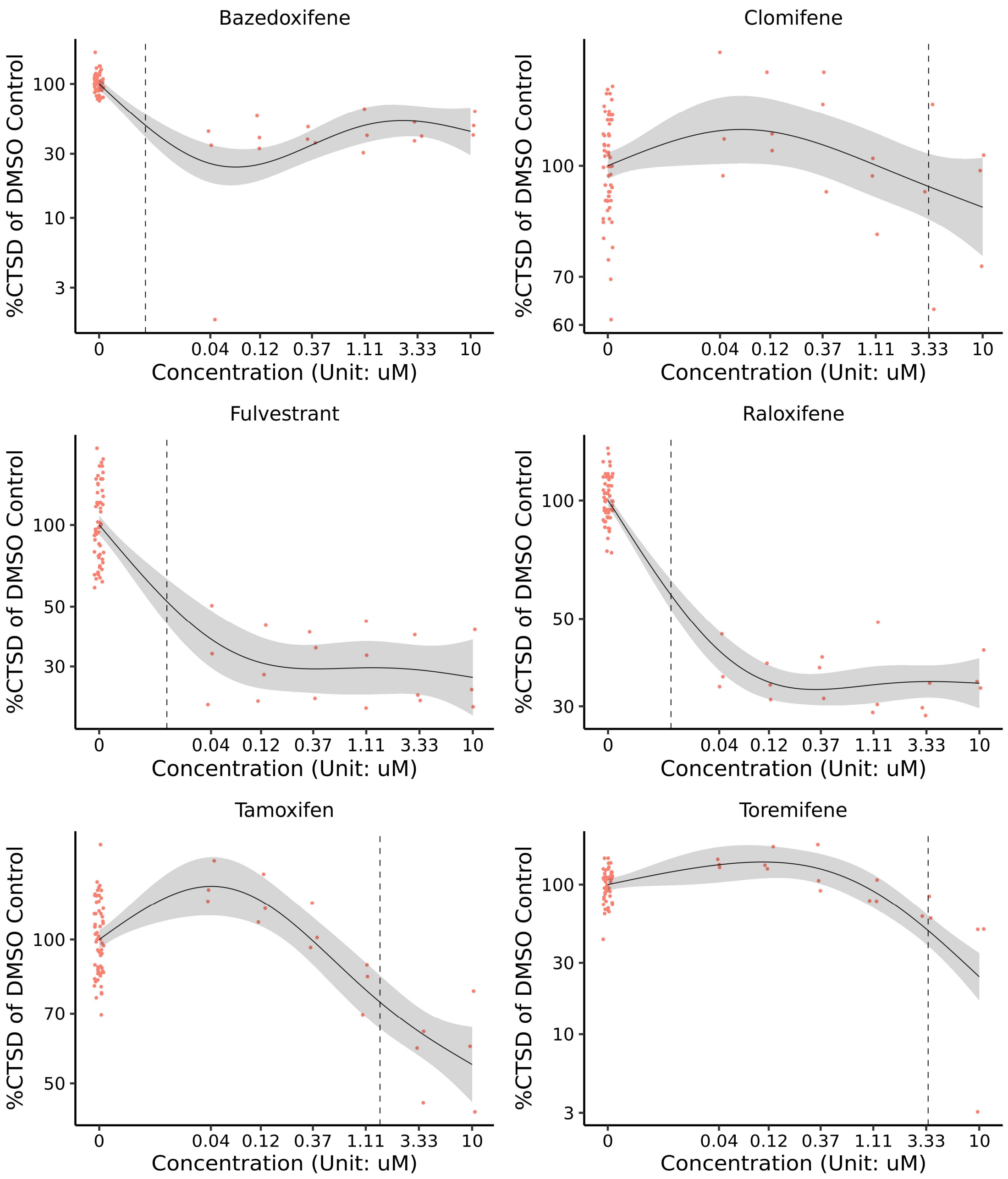

Supplementary Fig. 5. Dose response curves describing how the CTSD expression level responds to varying concentrations of bazedoxifene, clomifene, fulvestrant, raloxifene, tamoxifen, and toremifene in MCF-7 cells. Colored dots represent experimental observations, while the solid curve represents the model fit. IC_{50} is indicated by vertical dashed lines. Shaded area represents 95% confidence intervals.

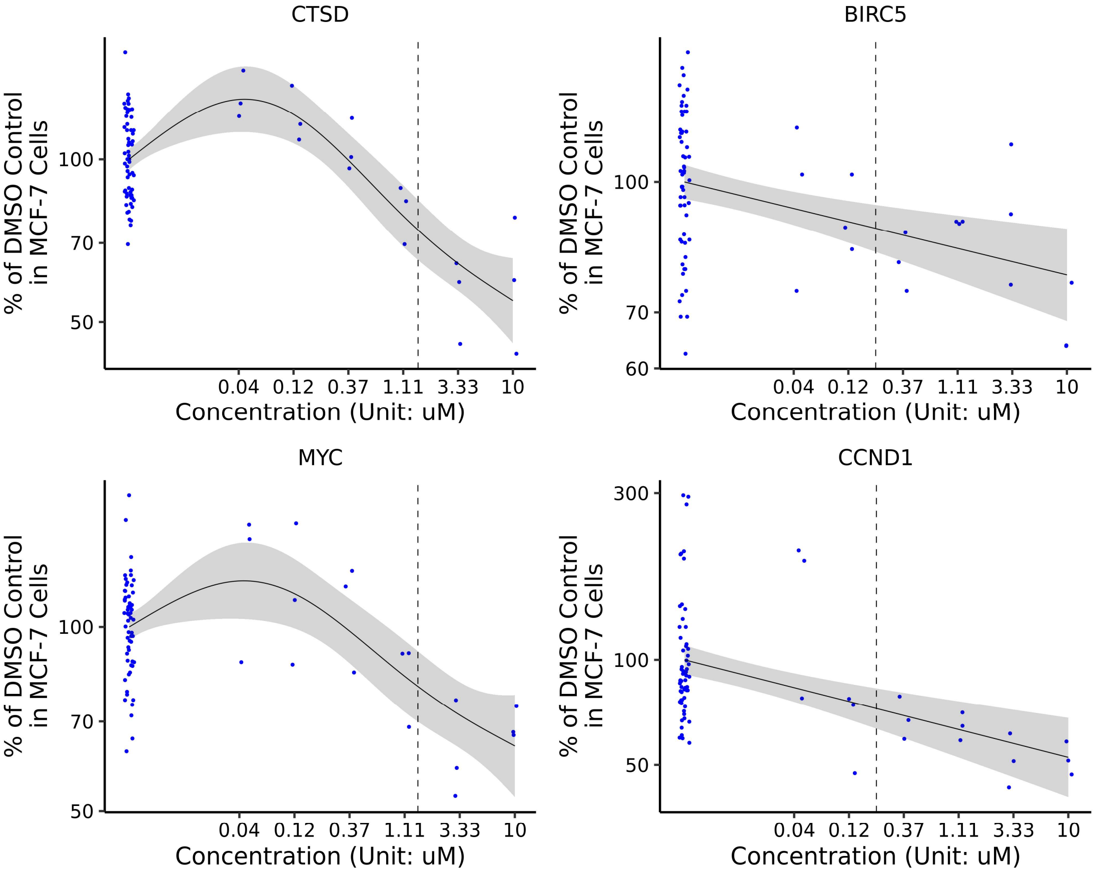

Supplementary Fig. 6. Dose response curves describing how the expression level of CTSD, BIRC5, MYC, and CCND1 responds to varying concentrations of tamoxifen in MCF-7 cells. Colored dots represent experimental observations, while the solid curve represents the model fit. IC_{50} is indicated by vertical dashed lines. Shaded area represents 95% confidence intervals.

Supplementary References

Edgar, R., M. Domrachev, and A. E. Lash. 2002. 'Gene Expression Omnibus: NCBI gene expression and hybridization array data repository', *Nucleic Acids Res*, 30: 207-10.

Koller, M. 2016. 'robustlmm: An R Package for Robust Estimation of Linear Mixed-Effects Models.', *J Stat Softw*, 75: 1-24.

Korotkevich, G., V. Sukhov, N. Budin, B. Shpak, M. N. Artyomov, and A. Sergushichev. 2016. "Fast gene set enrichment analysis." In. bioRxiv.

Subramanian, A., R. Narayan, S. M. Corsello, D. D. Peck, T. E. Natoli, X. Lu, J. Gould, J. F. Davis, A. A. Tubelli, J. K. Asiedu, D. L. Lahr, J. E. Hirschman, Z. Liu, M. Donahue, B. Julian, M. Khan, D. Wadden, I. C. Smith, D. Lam, A. Liberzon, C. Toder, M. Bagul, M. Orzechowski, O. M. Enache, F. Piccioni, S. A. Johnson, N. J. Lyons, A. H. Berger, A. F. Shamji, A. N. Brooks, A. Vrcic, C. Flynn, J. Rosains, D. Y. Takeda, R. Hu, D. Davison, J. Lamb, K. Ardlie, L. Hogstrom, P. Greenside, N. S. Gray, P. A. Clemons, S. Silver, X. Wu, W. N. Zhao, W. Read-Button, X. Wu, S. J. Haggarty, L. V. Ronco, J. S. Boehm, S. L. Schreiber, J. G. Doench, J. A. Bittker, D. E. Root, B. Wong, and T. R. Golub. 2017. 'A Next Generation Connectivity Map: L1000 Platform and the First 1,000,000 Profiles', *Cell*, 171: 1437-52 e17.

Subramanian, A., P. Tamayo, V. K. Mootha, S. Mukherjee, B. L. Ebert, M. A. Gillette, A. Paulovich, S. L. Pomeroy, T. R. Golub, E. S. Lander, and J. P. Mesirov. 2005. 'Gene set enrichment analysis: a knowledge-based approach for interpreting genome-wide expression profiles', *Proc Natl Acad Sci U S A*, 102: 15545-50.

Tang, J., Z. U. Tanoli, B. Ravikumar, Z. Alam, A. Rebane, M. Vaha-Koskela, G. Peddinti, A. J. van Adrichem, J. Wakkinen, A. Jaiswal, E. Karjalainen, P. Gautam, L. He, E. Parri, S. Khan, A. Gupta, M. Ali, L. Yetukuri, A. L. Gustavsson, B. Seashore-Ludlow, A. Hersey, A. R. Leach, J. P. Overington, G. Repasky, K. Wennerberg, and T. Aittokallio. 2018. 'Drug Target Commons: A Community Effort to Build a Consensus Knowledge Base for Drug-Target Interactions', *Cell Chem Biol*, 25: 224-29 e2.

Wang, K., J. Sun, S. Zhou, C. Wan, S. Qin, C. Li, L. He, and L. Yang. 2013. 'Prediction of drug-target interactions for drug repositioning only based on genomic expression similarity', *PLoS Comput Biol*, 9: e1003315.

Wood, Simon N. 2017. *Generalized additive models : an introduction with R* (CRC Press/Taylor & Francis Group: Boca Raton).
